# ﻿Neotypification for five names linked to *Arenaria* (Caryophyllaceae) for the endemic flora of Peru and Bolivia

**DOI:** 10.3897/phytokeys.230.107263

**Published:** 2023-08-07

**Authors:** Daniel B. Montesinos-Tubée, Duilio Iamonico

**Affiliations:** 1 Botanic Garden and Botanical Museum Berlin (BGBM), Freie Universität Berlin, Königin–Luise–Str. 6–8, 14195 Berlin, Germany Freie Universität Berlin Berlin Germany; 2 Instituto Científico Michael Owen Dillon, Av. Jorge Chávez 610, Cercado, Arequipa, Peru Instituto Científico Michael Owen Dillon Arequipa Peru; 3 Instituto de Ciencia y Gestión Ambiental de la Universidad Nacional de San Agustín de Arequipa. Calle San Agustín 108, Arequipa, Peru Instituto de Ciencia y Gestión Ambiental de la Universidad Nacional de San Agustín de Arequipa Arequipa Peru; 4 Department of Environmental Biology, University of Rome Sapienza, Piazzale Aldo Moro 5, 00185 Rome, Italy University of Rome Sapienza Rome Italy

**Keywords:** Arenarieae, Bolivia, nomenclatural type, Peru

## Abstract

The names *Arenariamattfeldii*, *A.pallens*, *A.peruviana*, *A.pintaudii*, and *A.stuebelii* (Caryophyllaceae, Arenarieae) from Peru and Bolivia were studied and neotypified based on specimens preserved at B and P.

## ﻿Introduction

*Arenaria* L. (Caryophyllaceae Juss.) is a genus comprising about 160–175 species of annual and perennial herbs mostly distributed in the northern temperate and subarctic regions, the Mediterranean, Mexico, and the Andes of South America (see Bittrich 1993; Hernández-Ledesma et al. 2015; POWO 2023) but other authors (Sadeghian et al. 2015) include 150–300 species in the genus. The molecular data by Greenberg and Donoghue (2011), Dillenberger and Kadereit (2014), and Sadeghian et al. (2015) showed that *Arenaria* is polyphyletic and several names were transferred to other genera (see e.g., Conti et al. 2014; Dillenberger and Kadereit 2014; Sadeghian et al. 2015; Iamonico 2016; De Luca et al. 2022). From a nomenclatural point of view, questions remaining to be addressed concern several names that are still untypified (see e.g., Iamonico 2013, 2014, 2016, 2019, 2022).

As part of ongoing studies on the systematics of Andean *Arenaria* (Montesinos-Tubée and Teillier 2022; Montesinos-Tubée and Iamonico 2023), here we present nomenclatural notes concerning some names in *Arenaria* described from the Andean region and included in section Dicranilla (Fenzl) F.N. Williams and sect. Leiosperma Williams.

## ﻿Materials and methods

The first author went to the type localities described by Weberbauer in Muschler (1911) and Macbride (1937) during expeditions carried out between 2015–2022 in the Central Andes and made numerous collections of *Arenaria*. The collections were deposited mostly at HSP and B. A subsequent exhaustive investigation of herbarium material at B (Herbarium Berolinense) revealed that some of the material appointed by Macbride (1937) was unfortunately not photographed prior to the destructions of large parts of the herbarium. Only an image of *Arenariamattfeldii* is available at the Berlin Negatives Digitization Project (https://collections–botany.fieldmuseum.org/project/6454).

The present study is based on the analysis of relevant literature (protologues included) and examination (mainly by the first author) of specimens preserved at the herbaria B, F, GOET, LPZ, MO, and P (acronyms according to Index Herbariorum - Thiers 2023).

The International Code of Nomenclature articles cited throughout the text follow the Shenzhen Code (ICN; Turland et al. 2018).

## ﻿Results and discussion

### ﻿ Arenaria mattfeldii

Taxon classificationPlantaeCaryophyllalesCaryophyllaceae

Baehni, Publ. Field Mus. Nat. Hist., Bot. Ser. 13(2/2): 601. 1937.

1CA2A251-B958-5F70-920C-94327A44AD21

#### Neotype

**(designated here).** Peru. La Libertad: Llautabamba, Huamachuco, 4650 m, 8 May 1954, *J. Infantes 4672* (B100747343!, Fig. 2).

**Figure 1.**
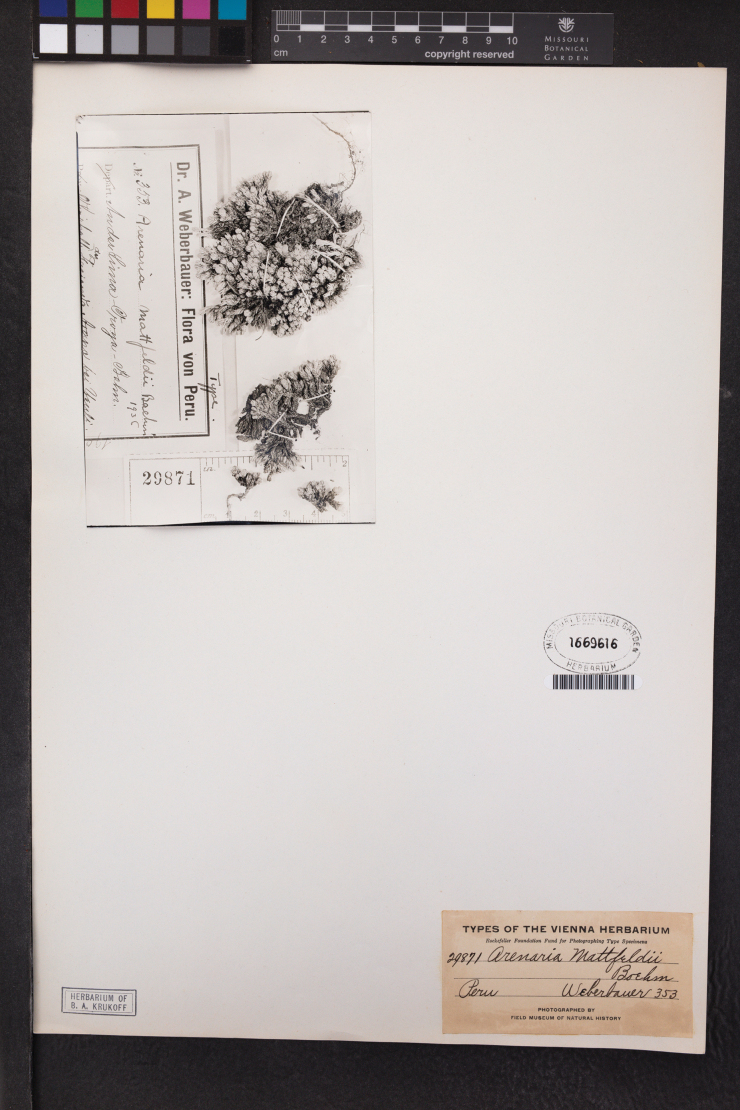
MO sheet of the type photograph of *Arenariamattfeldii* [Weberbauer 353].

Macbride (1937: 601) validly published *Arenariamattfeldii* providing a detailed diagnosis accompanied by the following note: “Junín: Yauli, above the Hacienda Arapa, near the Lima–Oroya railroad, *Weberbauer 353* (type, in Herb. Deless.)”. “Herb. Deless.” means “Herbarium Delessert”. According to HUH–Index of Botanists (2013a), the surname “Delessert” refers to, at least, three different people, i.e. Adolphe Delessert (collector in India, Indonesia, Malaysia, Reunion), Henri Delessert (collector in Cuba), and Jules Paul Benjamin Delessert (collector in Brazil). Unfortunately, Macbride (1936: 9–81), in the Introduction of his *Flora of Peru*, did not mention “Delessert” and we did not find any other reference to an “Herb. Deless.” throughout the various volumes of the flora. However, based on the collector reported by Baehni in Macbride (1937: 601), i.e., Weberbauer, we tried to search original material at B, where Weberbauer’s collection is preserved (HUH–Index of Botanists 2013b). Unfortunately, no specimen could be traced (the section holding these collections at herbarium B was destroyed during the II World War). No other specimen useful for the purpose of lectotypification was found. We traced only a photograph (no. 29871) of the original Weberbauer’s collection no. 353 at F and MO (Fig. 1). However, this photograph, which was made after 1937, cannot be considered as part of the original material for *A.mattfeldii* under the Art. 9.4a of ICN; hence a neotypification is required under the Art. 9.8 of the ICN. We here designate as a neotype, a specimen collected in Peru and preserved at B (B100747308).

**Figure 2.**
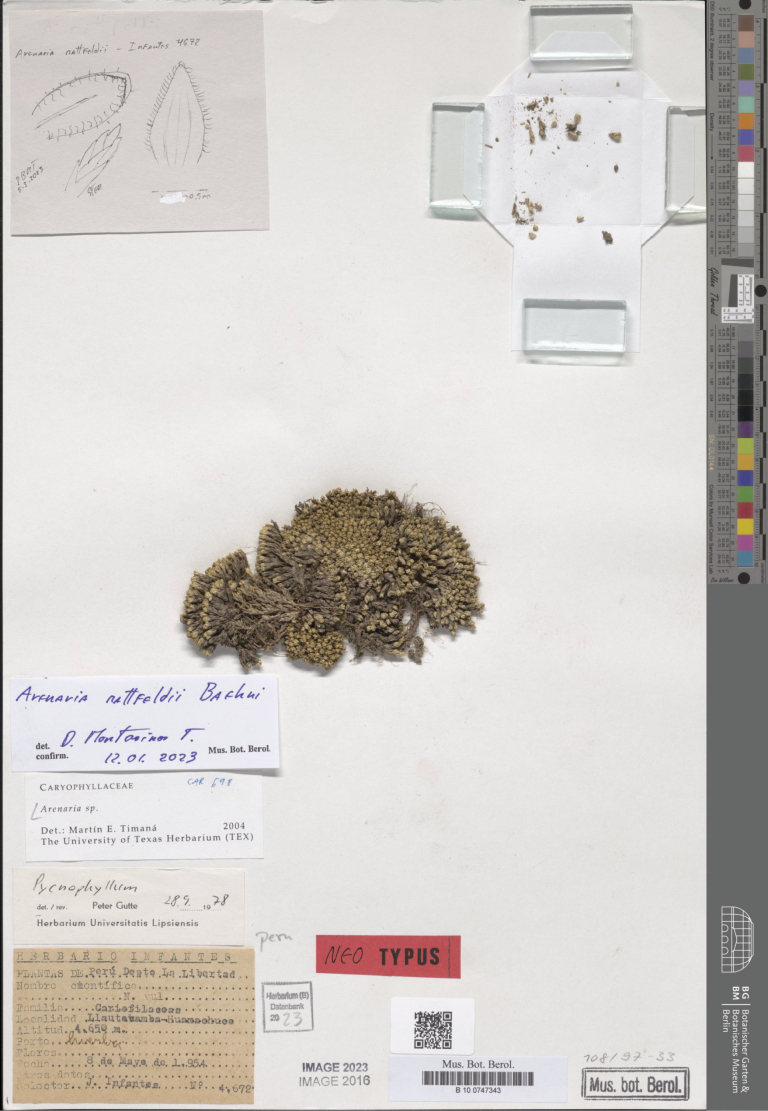
Neotype of *Arenariamattfeldii* Baehni (*J. Infantes 4672*, B100747308).

#### Observations.

The species is considered narrowly endemic to Junín and designated as Critically Endangered by Cano and Sánchez (2006).

#### Description.

Perennial herb, densely caespitose, matt-forming, 3–5 cm height, 10 cm wide; leaves 1.5–2.0 mm long, closely imbricate, shortly appressed, thick, lamina with deltate to broadly ovate, apex obtuse or rarely acute, base truncate; margins shortly revolute and ciliate, trichomes simple, 0.10–0.15 mm long and irregularly shaped, rarely straight, leaf lamina surface glabrous both on the upperside and underside, and without a visible midrib; flowers apical, pedicels < 1 mm long, sepals 2 mm long, ovate-deltate, apex slightly curved downwards; petals reduced, ovate, translucid, ½ as long as sepals; stamens 10, ovary 0.75–1.00 mm long, glabrous, styles two; seeds about 8, with smooth surface.

#### Specimens examined.

**Peru. Junín**: La Oroya, Morococha, Hacienda Pucará, 4700 m., 23 May 1974, *P. Gutte 2353b* (LPZ!).

### ﻿ Arenaria pallens

Taxon classificationPlantaeCaryophyllalesCaryophyllaceae

Muschl., Botanische Jahrbücher für Systematik, Pflanzengeschichte und Pflanzengeographie 45(4): 450–451. 1911.

D8D16277-31B6-5AFD-99F8-54C5ECF96479

#### Neotype

**Figure 3.**
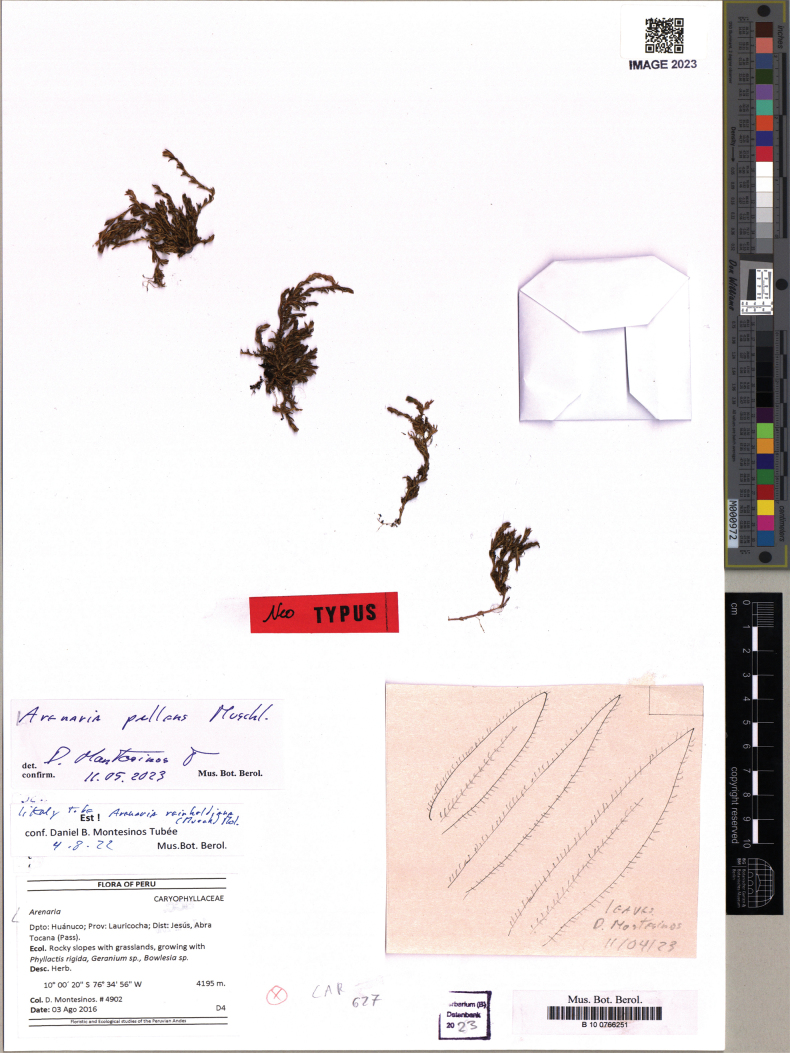
Neotype of *Arenariapallens* Muschl. (*D. Montesinos 4902*, B100766251).

**(designated here).** Peru. Huánuco: Lauricocha, Jesús, Abra Tocana, 4195 m, 03 Aug 2016, *D. Montesinos 4902* (B–100766251!, Fig. 3).

Muschler (1911: 450–451) validly published the name *Arenariapallens*, giving a detailed description, as well as the citation of a syntype (Art. 9.6 of ICN), i.e., “Peru: ad viam ferream inter oppida Lima et Oroya ad hacienda Arapa prope Yauli, ad rupes, in 4400 m altitudine” (Weberbauer 267). –Specimina florigera fructiferaque 18–27 Januarii 1902 – Herb. Berol. [Herbarium Berolinensis, B]”. According to Hiepko (1987), the only known collection at B was destroyed. Consequently, lacking original material (Art. 9.3 of ICN), a neotypification is required. The analysis of two specimens stored at B revealed them as alternative material from near the type locality, which is here chosen as neotypes. Note that Muschler’s taxon is a member of Arenariasect.Dicranilla which has the characteristic ciliate leaf margins and bisexual flowers (see also Timaná, 2017) as typical for *A.pallens*. We here designate a collection made by one of the authors (DBMT) as the neotype of Muschler’s *A.pallens*. The species is considered narrowly endemic and was designated as Critically Endangered by Cano and Sánchez (2006). The finding of the species in the Huánuco department expands its distribution and it can be considered to occur in the Pasco department as well and Junín department as first established by Cano and Sánchez (2006).

Further analysis needs to be made for three species described by Muschler (1911) which were not found at the explored localities, in addition to the lack of herbarium material, types destroyed and doubtful protologues. It is concluded that Muschler (1911) described the taxa with an apparent mixture of characters (Ryding 1939) as seem to occur in *Arenariahorizontalis* (Muschl.) Molinari (= *Pycnophyllumhorizontale* Muschl.), due to impossible character symmetry as described on page 449, a fact that was not corrected by Molinari (2016).

#### Description.

Diffuse herb, with branches of about 2–8 cm long, more or less angled and pubescent near the nodes but glabrous in mid-sections; internodes of about 6–10 mm long; leaves linear to linear-lanceolate, rarely ovate-lanceolate, thick or fleshy, with acuminate apex bearing a mucronate tip, base petiolate, lamina of about 5–10 mm long × 1.0–3.0(–3.5) mm width, bearing trichomes at the base, margins and apex; pedicels filiform, densely pubescent or rarely glabrous, less than 1 cm long, curved when fruiting; sepals ovate-oblong, carinate, apex acuminate, hirsute or puberulent along the margins, 3–4 mm long; petals tending to be shorter than the sepals, ovate-oblong and obtuse; anthers pale yellow; styles 3; capsule longer than the calyx; 8 seeds, shiny and smooth.

#### Specimens examined.

**Peru. Huánuco**: Huamalies, Singa, W of Bellas Flores, 3601 m, 26 jul 2016, *D. Montesinos 4853* (B–1007613519!).

### ﻿ Arenaria peruviana

Taxon classificationPlantaeCaryophyllalesCaryophyllaceae

(Muschl.) Molinari, Polish Bot. J. 61(2): 276. 2016

8DD95235-0BA9-5DBF-B868-9228A28252D4

≡ Pycnophyllumperuvianum Muschl., Bot. Jahrb. Syst. 45(4): 457–458. 1911.

#### Neotype

**Figure 4.**
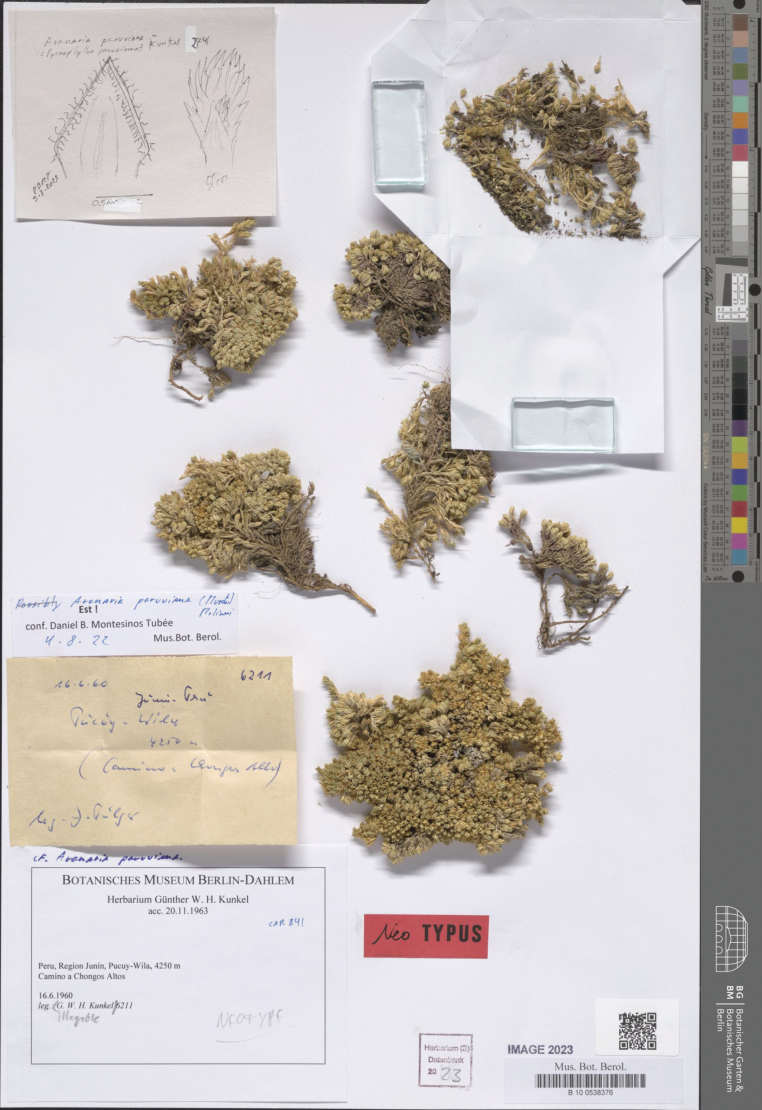
Neotype of *Arenariaperuviana* (Muschl.) Molinari (*G.W.H. Kunkel 6211*, B100538376).

**(designated here).** Peru. Junín: Pacuy-Wila, 4250 m, 16 Jun 1960, *G.W.H. Kunkel 6211* (B100538376!, Fig. 4).

Muschler (1911: 276) validly published *Pycnophyllumperuvianum* with a detailed description; including the provenance and habitat reported as “Peru: Prope La Oroya in departamento Junin, in formation planti caespitosis ac pulviniaribus composite, 4300 m s. m.”, as well as the collector and number of the original collection (“Weberbauer 2597”) followed by “Specimina florigera fructiferaque Februario 1903. – Herb. Berol.”. The only known collection of *Pycnophyllumperuvianum* by Weberbauer at B (“Herb. Berol.” = Herbarium Berolinensis) was destroyed (see Hiepko 1987). The cited collection by Molinari (2016) at MOL (where additional material of Weberbauer is preserved; see HUH–Index of Botanists 2013b) is inexistent (pers. observ.; see also Timaná 2017). Lacking original material, a lectotype cannot be designated (Arts. 9.3 and 9.4 of ICN) and a neotypification is required (Art. 9.8 of ICN). Despite knowing this, Molinari (2016) published the new combination without observing or selecting a type specimen (Timaná 2017). We consider a collection made by Kunkel nearby the locus classicus as the neotype of the name *Pycnophyllumperuvianum*.

#### Observations.

Muschler’s taxon is a member of Arenariasect.Dicranilla which has the characteristic of having ciliate leaves and bisexual flowers (see also Timaná, 2017). The species is considered as narrowly endemic and treated as Critically Endangered by Cano and Sánchez (2006).

#### Description

**(see also Muschler 1911 and Macbride 1937).** perennial herb, densely caespitose, 3–5 cm long × 15 cm width; densely leaved, leaves 1.2–1.8 mm long, imbricate, shortly appressed, thick, lamina subtriangular in outline, rigid or fleshy, densely ciliate, apex acuminate; margins ciliate and shortly revolute, trichomes long and thin, shortly distant at the base, ca. 0.2 mm long; flowers with short pedicels, less than 0.5 mm long, sepals 2 mm long, linear to ovate-lanceolate, scarious and with an acute apex; petals absent; stamens 2–3 mm long, style slightly longer; seeds triangular and compressed, shiny.

### ﻿ Arenaria pintaudii

Taxon classificationPlantaeCaryophyllalesCaryophyllaceae

Molinari, Polish Bot. J. 61(2): 275. 2016, nom. nov. pro

4E9A45F6-1082-5261-9E6A-D34B3B802EB4

Alsine rupestris Muschl., Bot. Jahrb. Syst. 45(4): 448–449. 1911, non Fenzl (1833).

#### Neotype

**Figure 5.**
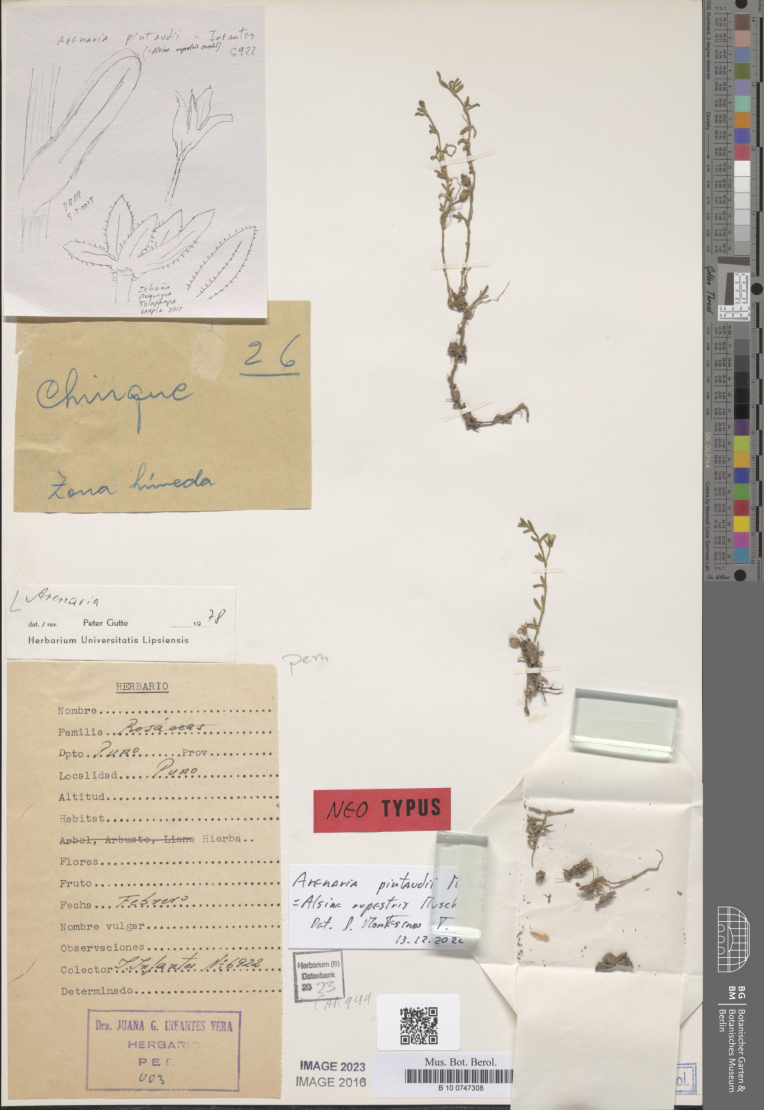
Neotype of *Arenariapintaudii* Molinari (*J. Infantes 6922*, B100747343).

**(designated here).** Peru. Puno: Puno, *J. Infantes 6922* (B–100747308, Fig. 5).

The name *Arenariapintaudii* was validly published by Molinari (2016: 275) as a *nomen novum pro Alsinerupestris* Muschler. Muschler (1911: 448–449), although validly published *A.rupestris*, overlooked the previous legitimate name *S.rupestris* (Scop.) Fenzl. [published in 1883 and currently accepted as *Facchiniarupestris* (Scop.) Dillenb. & Kadereit]. Hence Muschler’s *Alsinerupestris* is illegitimate (later homonym) according to the Art. 53.1 of ICN. According to Art. 7.4 of ICN, “A replacement name ... is typified by the type of the replaced synonym”. Therefore, the typification of Molinari’s *Arenariapintaudii* must be made studying Muschler’s *Alsinerupestris*. Muschler (1911) provided a detailed description, the provenance and habitat (“Peruvia: supra Ananca, in Sandia provincial, rupiphus, 5100 ms.m.”), collector and number of collection (“Weberbauer # 1042”); he also reported: “Specimina florigera fructiferaque 16 Mai 1902. – Herb. Berol. [Herbarium Berolinense, now B]”. The only known collection of *Alsinerupestris* at B was destroyed according to Hiepko (1987) and no further original material could be found. It remains unclear how Molinari (2016) published the new name without observing or selecting a type specimen. Anyway, a neotypification is required under the Art. 9.8 of ICN.

#### Observations.

The species is considered as Critically Endangered (Cano and Sánchez 2006) and it seems to have well-established populations in certain sectors of the altiplano in the department of Puno according to herbarium labels and personal observations. Moreover, few populations were observed north of the department of Moquegua from where one of the additional observations comes from.

#### Description.

Pulvinate herb with several branches, 3–15 cm long, decumbent or procumbent, glabrous, internodes ca. 1 cm long; leaves lanceolate to ovate in outline, bearing an amplexicaul base and acute apex, 8–12 mm long × 2–3 mm wide, margins densely ciliated, rarely glabrous; pedicels up to 5 mm long, erect or curved, puberulent or glabrous; sepals oblong, 3.5–5.0 mm long; petals ovate-cuneate, ca. 4.5 mm long; seeds smooth, blackish.

#### Specimens examined.

**Peru. Puno**: Santa Lucia, 3600 m, Nov 1939, *J.E. Sharpe 94* (K!); **Puno**: Santa Lucia, 3600 m, Nov 1939, *J.E. Sharpe 107* (K!); **Puno**: Puno, *J. Infantes 6922* (B100747308); **Puno**: Santa Lucia, 3600 m, 10 Nov 1939, *J.E. Sharpe 94* (K!); **Puno**: Azángaro, Arapa, 3820 m, 17 Feb 1948, *P. Aguilar 100* (USM–18587!); **Puno**: Cerro entre rocas, 3900 m, 11 Feb 1948, *P. Aguilar 148* (USM–18576!); **Moquegua**: Ichuña, Tolapampa, 4040 m, 14 Apr 2012, *D. Montesinos & F. Calizaya 3823a* (B–101156477!).

### ﻿ Arenaria stuebelii

Taxon classificationPlantaeCaryophyllalesCaryophyllaceae

Hieron., Botanische Jahrbücher für Systematik, Pflanzengeschichte und Pflanzengeographie 21(3): 307. 1895.

C42E67FC-C153-58B6-9AB2-7FF01355A601

#### Neotype

**Figure 6.**
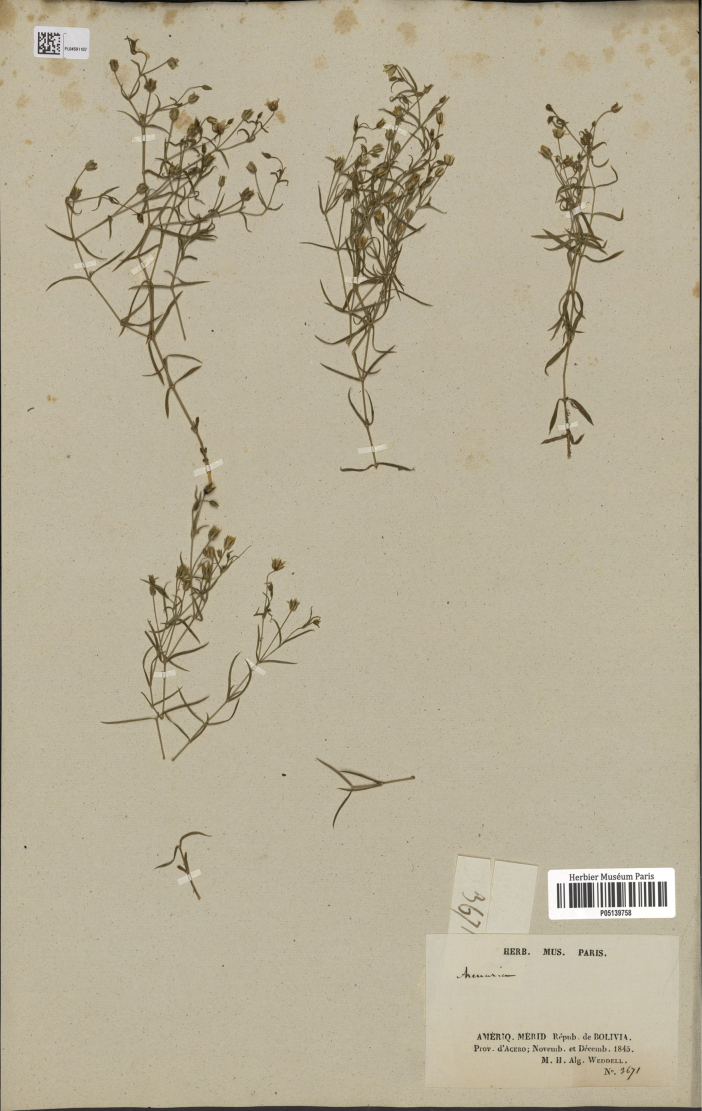
Neotype of *Arenariastuebelii* Hieron. (*M.H. Weddell 3691*, P05139758).

**(designated here). Bolivia. Hernando Siles**: Azero, Nov–Dec 1845, *M.H. Weddell 3691* (P–05139758!, Fig. 6, http://mediaphoto.mnhn.fr/media/1441409343048mmKRKfSJcLXpH0CF).

Hieronymus (1895: 307) provided a detailed description of *Arenariastuebelii*, citing the following syntype (Art. 9.6 of ICN): “Bolivia: crescit locis aridis Puna dictis supra Taca in valle Yungas, ubi floret mense dicembri (coll. boliv. n. 48d)”. We have been unable to locate a specimen collected by M.A. Stüebel (the botanists who Hieronymus dedicated the species) and numbered as 48d at B (where Stüebel’s herbarium and type are preserved; HUH–Index of Botanists 2013c), since they were mostly destroyed during the II World War. After an exhaustive investigation of different plant material collected near locus classicus, we here decide to designate a specimen from P (barcode P–05139758) as the neotype of the name *Arenariastuebelii*.

#### Description.

Herb with numerous stems branching from the taproot, 10–30 cm long, ascending and covered with minute trichomes varying from 0.1–0.2 mm long; leaves linear to narrowly linear-lanceolate, acute or acuminate, pubescent on the adaxial and abaxial sides, lamina ca. 8 mm × 1 mm; flowers with long pedicels, puberulent or scarious; sepals ovate, with scarious margins, 3–4 mm long × 2 mm wide; petals white, ovate-oblong, obtuse, 6–7 mm long × 3 mm wide; seeds globose, flattened, black, smooth and shiny.

#### Specimens examined.

**Bolivia. Tarija**: Arce, Municipio Padcaya, Reserva Nacional de flora y fauna Tariquía, 2457 m, 27 Apr 2005, *M. Serrano et al. 6327* (MO–5956858!); **Franz Tamayo**: Parque Nacional Madidi, 2810 m., 23 Jun 2005, *A. Fuentes & E. Cuevas 8622* (MO–5956863!); **La Paz**: Sud Yungas, Chulumani, 1972 m, 27 jun 2007, *D. Ibañez & R. Hurtado 603* (B–100720358!); **La Paz**: Sud Yungas, Yanacachi a la Chojlla, 2100 m, 7 Sep 1987, *E. Vargas & R. Seidel 496* (B–100720356!).

## Supplementary Material

XML Treatment for Arenaria mattfeldii

XML Treatment for Arenaria pallens

XML Treatment for Arenaria peruviana

XML Treatment for Arenaria pintaudii

XML Treatment for Arenaria stuebelii

## ﻿Additional information

### Author ORCIDs

Daniel B. Montesinos-Tubée https://orcid.org/0000-0002-4439-5089

Duilio Iamonico https://orcid.org/0000-0001-5491-7568

### Data availability

All of the data that support the findings of this study are available in the main text.
